# Intestinal Microbiota Composition in Patients with Type 2 Diabetes and Effects of Oral Antidiabetics

**DOI:** 10.3390/jcm14082786

**Published:** 2025-04-17

**Authors:** Ahmet Toygar Kalkan, Goknur Yorulmaz, Aysen Akalin, Ener Cagri Dinleyici

**Affiliations:** 1 Faculty of Medicine, Department of Endocrinology, Eskisehir Osmangazi University, Eskisehir 26040, Türkiye; 2 Faculty of Medicine, Department of Pediatrics, Eskisehir Osmangazi University, Eskisehir 26040, Türkiye; timboothtr@yahoo.com

**Keywords:** type 2 diabetes, diabetes mellitus, microbiota, metformin, linagliptin

## Abstract

**Introduction**: The cause–effect relationships between microbiota composition changes and type 2 diabetes (T2D) are complex, likely involving two-way interactions, and require further elucidation. Few studies have examined the interactions of antidiabetic drugs with the gut microbiota. This study’s goal was to evaluate the gut microbiota of patients with type 2 diabetes at first diagnosis and again after 12 weeks of taking oral antidiabetic drugs. **Methods**: We performed a fecal microbiota analysis of adult patients who recently received a T2D diagnosis and healthy adults. We compared the microbiota compositions between the T2D patients and healthy controls; we also evaluated changes from baseline to 12 weeks of treatment in the total group receiving oral antidiabetics, as well as in the subgroups receiving metformin and linagliptin. **Results**: The alpha diversity and beta diversity indices were different at baseline between patients with type 2 diabetes and healthy controls. The LEfSe analysis showed that, at the genus level, the *Lactobacillus*, *Rothia*, *Collinsella*, and *Eubacterium* genera increased in relative abundance in the T2D group while, at the species level, the *Rothia mucilaginosa*, *Collinsella aerofaciens*, and *Eubacterium bioforme* strains were found to be dominant in the T2D group. *Faecalibacterium* at the genus level and *Faecalibacterium prausnitzii* at the strain level increased in relative abundance in the T2D group after 12 weeks. After 12 weeks of intervention, the alpha diversity indices were significantly lower in the T2D group compared to the control group. At the end of the 12th week, the *Gemmiger* and *Collinsella* genera were dominant in the T2D group, with *Gemmiger formicilis* and *Collinsella aerofaciens* being dominant at the species level; in the control group, *Bacteroides* and *Alistipes* were dominant at the genus level, and *Prevotella stercorea* and *Alistipes finegoldii* were dominant. There was no difference in the LEfSe analysis results between baseline and 12 weeks of linagliptin treatment. At the strain level, *Gemmiger formicilis*, *Ruminococcus bromii*, *Rothia mucilaginosa*, and *Lactobacillus ruminis* were predominant at the start of metformin treatment; however, after 12 weeks, *Collinsella aerofaciens* became predominant. **Conclusions**: We report that there is a substantial change in the composition of the gut microbiota in patients with T2D. Oral antidiabetic treatments, especially metformin, have some beneficial effects on microbiota composition. Few studies have explored the interaction of antidiabetic drugs with the gut microbiota; further research will elucidate the clinical impact of these changes in gut microbiota composition in diabetes.

## 1. Introduction

The human gut microbiota has recently emerged as having a significant influence on various diseases, particularly non-communicable diseases [1]. The gut microbiota has been widely associated with the onset of obesity and its related conditions, including type 2 diabetes (T2D) and insulin resistance [2]. Furthermore, data indicate that T2D can alter the composition of the gut microbiota. The causal links between dysbiosis and T2D are intricate and require further clarification. They likely form a bidirectional interaction [3,4,5]. A crucial aspect to consider when examining the involvement of the gut microbiota in T2D is the potential confounding influence of diabetes medications [6,7,8]. Antidiabetic drugs may influence the gut microbiota to enhance glucose homeostasis; hence, the gut microbiota could further impact the effectiveness of pharmacological treatment [3,9]. Recent studies suggest that the gut microbiota mediates the glucose-lowering effects of metformin, influencing the composition and functionality of the microbiome and augmenting the alterations associated with T2D [10,11,12]. Metformin enhances glucose homeostasis by modulating the gut microbiota, potentially exerting glucose-lowering effects through various mechanisms, including regulating intestinal glucose absorption, promoting the proliferation of short-chain fatty acid-producing bacteria that generate butyrate and propionate, stimulating the growth of mucin-degrading bacteria, augmenting the secretion of the gut hormone glucagone-like peptide-1 (GLP-1), regulating bile acid metabolism, and preserving the integrity of the intestinal barrier [3,10].

Besides metformin, various antidiabetic medications with distinct mechanisms of action are available. Limited research has investigated the relationships between these various antidiabetic medications and the gut microbiota [3,13,14]. With results comparable to those for metformin, existing research has suggested that the gut microbiota may serve as a potential modulator of these drugs. Dipeptidyl peptidase-4 (DPP-4) inhibitors, a new category of oral medications for T2D management, can successfully synergize with existing antidiabetic therapies to attain blood glucose targets [15]. There is limited information about the effects of DPP-4 inhibitors on microbiota composition [16,17].

In this study, we aimed to evaluate the intestinal microbiota composition at baseline and at week 12 in subjects diagnosed with T2D, receiving metformin or linagliptin, and to compare the groups within themselves and with healthy controls.

## 2. Materials and Methods

This was a case–control study involving patients with T2D, conducted from 2020 to 2021 at the Endocrinology and Metabolism Unit of Eskisehir Osmangazi University Faculty of Medicine in Türkiye. This research received financial support from the Eskişehir Osmangazi University Scientific Research Projects (TSA-2021-1587).

### 2.1. Inclusion and Exclusion Criteria

This study was planned to include adult patients older than 18 years with T2D and HbA1c levels of ≥6.5 and ≤10.0%, who had not received any glucose-lowering drug in the previous 12 weeks, who were followed up in the Endocrinology Department. We followed up with the patients with a single antidiabetic drug for a period of three months. Children, adolescents, and those with a body mass index (BMI) of >40, previous use of antibiotics or probiotics within the last 8 weeks, inflammatory or functional bowel disease, a history of gastrointestinal cancer or surgery (including bariatric surgery), acute or severe gastrointestinal symptoms requiring medical treatment, or a previous history of acute coronary syndrome or stroke were excluded from this study. The control group comprised persons, matched in age and gender to the research group, who were healthy, had not used antibiotics or probiotics in the preceding eight weeks and met the inclusion criteria. The serum HbA1c levels in the control group were within the normal limit.

We noted the patients’ age, gender, and clinical characteristics after obtaining their consent. The study team organized their treatment based on their disease status; eligible patients received metformin or linagliptin. The same study team in the Division of Endocrinology closely monitored the patients and performed clinical evaluations at the end of the 12th week. The study team asked patients to contact them if they needed any medication, especially antibiotics, during the 12-week period. Patients also received a diet and exercise counseling plan. The metformin dose was 2000 mg/day for 12 weeks of monotherapy, and the linagliptin dose was 5 mg once daily for 12 weeks of monotherapy.

### 2.2. Endpoints of This Study

The primary endpoint of this study was to evaluate the change in microbiota composition in T2D patients from baseline to the end of the 12th week and to compare this change with that in healthy controls. The secondary endpoint involved comparing the microbiota compositions of patients with those of healthy controls both at baseline and at the end of the 12th week. Additionally, it involved comparing the changes in intestinal microbiota composition after 12 weeks of metformin treatment or 12 weeks of linagliptin treatment to those observed for the healthy controls.

### 2.3. Fecal Samples and Microbiota Analysis

At least 5 mL of fresh stool sample was collected on Day 0 of this study and again 12 weeks later in a 15 mL Falcon tube. It was quickly frozen at −80 °C and kept upright until DNA extraction. At the end of the extraction process, an average of 50–60 ng of genomic DNA was obtained, diluted with 200 µL of CDT (elution buffer). We performed bacterial 16S ribosomal RNA (rRNA) gene target sequencing from the genetic materials obtained in our study. We amplified the genomic DNA using 16S V3-V4 314F-860R primer sets and prepared the libraries using the Nextera XT DNA library preparation kit and indexes (Illumina, San Diego, CA, USA). We cleaned the Amplicon libraries using AMPure XP from Beckman Coulter, carefully selecting large fragments. After preparing the library, we ran the sequencing on a NovoSeq 6000 (Illumina).

### 2.4. Bioinformatic and Statistical Analysis

We transferred paired-end Illumina reads (2 × 250) to Qiime2 media [18]. All the samples had an array depth exceeding 100X, and we did not exclude any sample from this study. Amplicon sequences with a low Phred score (<Q30) and chimeric amplicon sequences were identified and removed from the system via the Qiime2 Dada2 program (q2-dada2). We mapped the Amplicon Sequence Variants (ASVs) generated by Dada2 to the GreenGenes (/greengenes.lbl.gov (accessed on 8 February 2022)) database [19,20]. The R 4.1 environment created the Phyloseq object from the qiime2 structure files [21,22]. We interpreted the alpha diversity assessment, which evaluates the diversity of taxonomic units of interest in a sample, using three different indices: Chao1, Shannon, and Simpson. We calculated the *p*-values between groups using the Kruskal–Wallis test. We used a beta diversity analysis to assess taxonomic differences between individuals based on Jaccard, Bray–Curtis, weighted UniFrac, and unweighted UniFrac calculations. We determined specific differences between groups using a differential abundance analysis and the Deseq2 R package [23]. A Linear Discriminant Analysis Effect Size (LEFSe) analysis was used to find statistically significant (LDA threshold value > 2, *p* < 0.05) taxonomies among the groups [24]. We used the JASP package program to statistically analyze the patient and control groups’ demographic and laboratory characteristics. Spearman correlation tests were performed for the correlation between serum HbA1c levels and alpha diversity indices. *p* < 0.05 was considered to indicate statistical significance.

## 3. Results

In this case–control study, 44 T2D patients who met the inclusion criteria were included. Twelve patients were excluded because of the combined treatment requirement. Regarding the oral antidiabetic agent, the study population was subdivided into groups receiving linagliptin (n = 16) or metformin (n = 16). Some patients were excluded from the full analysis set due to protocol violations (not completing 12 weeks of treatment: metformin, n = 5; linagliptin, n = 3). After 12 weeks of follow-up, 24 T2D patients (14 men, 10 women, mean age 54.5 ± 8.7 years, 42–72 years) were considered eligible for the post-treatment evaluation. The control group consisted of 10 age- and gender-matched healthy adults (5 men, 5 women; mean age 50.9 ± 5.0 years, 45–61 years). The demographic and clinical characteristics were similar between the metformin and linagliptin groups and the healthy controls at baseline.

At baseline, the mean HbA1c levels were 8.21 ± 1.71, min–max 6.7–9.9, in the patient group. At 12 weeks of intervention, the mean HbA1c levels were significantly lower (6.81 ± 0.78, min–max 5.7–8.5) in the T2D group compared to the baseline group (*p* < 0.001). The 24 T2D patients included in this study were then evaluated separately with regard to the treatment received. Among them, 13 patients (7 men, 6 women; mean age 57.3 ± 10.1 years, 42–72 years) were treated with linagliptin, and 11 patients (7 men, 4 women; mean age 51.0 ± 4.7 years, 45–61 years) were treated with metformin as monotherapy oral antidiabetics. At baseline, the mean HbA1c level was 7.53 ± 1.29 (min–max 5.9–9.8) in the linagliptin group and 8.59 ± 1.06 (min–max 6.2–10) in the metformin group. At 12 weeks of intervention, the mean HbA1c level was 6.95 ± 0.94 (min–max 5.7–8.5) in the linagliptin group and 6.64 ± 0.52 (min–max 5.85–7.6) in the metformin group. The serum HbA1c levels were significantly lower at 12 weeks of intervention compared with the baseline levels for both the linagliptin and metformin groups (*p* < 0.05 and *p* < 0.01, respectively).

### 3.1. Comparison of Microbiota Compositions of T2D Patients and Healthy Controls Before Treatment (Baseline)

In our study, the observed OTUs, Chao1 index, Simpson index, and Shannon index were used to evaluate the alpha diversity. At baseline, the median value of the Chao1 index was lower (*p* < 0.05), and the Shannon index was lower without statistical significance in the T2D group than in the control group. The median number of observed OTUs in the T2D group was lower than that in the control group (*p* < 0.05). The Bray–Curtis, Jaccard, weighted UniFrac, and unweighted UniFrac principal coordinate analysis (PCoA) results for T2D patients were different from those for the control group (*p* < 0.05). There was no correlation between the HbA1c levels and alpha diversity index parameters (*p* > 0.05).

**Figure 1:**
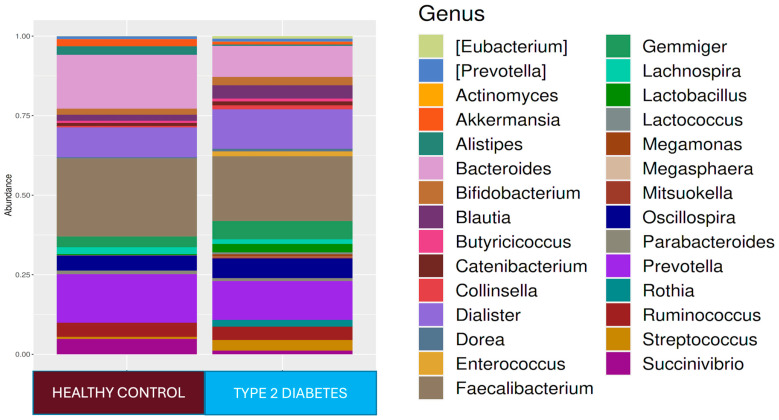

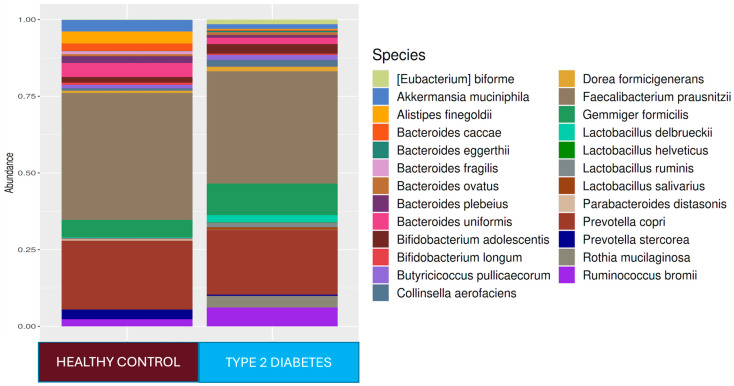
Comparison of fecal microbiota compositions of healthy controls and patients with type 2 diabetes at the genus and species levels at admission. Maroon: healthy controls. Blue: T2D.

At the phylum level, *Firmicutes* 61.4%, *Bacteroidetes* 27.4%, *Proteobacteria* 5.9%, *Actinobacteria* 2.7%, and *Verrucomicrobia* 1.4% were found in the T2D group, while *Firmicutes* 73.6%, *Bacteroidetes* 17.4%, *Proteobacteria* 4.0%, and *Actinobacteria* 3.4% were found in the healthy adult group. While the *Firmicutes* phylum was predominant in healthy adults (*p* < 0.001), *Bacteroidetes* (*p* < 0.001) and *Tenericutes* (*p* < 0.001) were higher in relative abundance in the T2D group compared to healthy adults. At the genus level, *Faecalibacterium* 18.2%, *Prevotella* 11.4%, *Dialister* 11.2%, *Bacteroides* 10.2%, *Oscillospira* 7.5%, *Ruminococcus* 5%, and *Gemmiger* 5% were dominant in the T2D group, while *Faecalibacterium* 22.1%, *Bacteroides* 16.5%, *Prevotella* 14.2%, *Dialister* 8.3%, *Oscillospira* 6.4%, *Ruminococcus* 5.1%, *Succinovibrio* 4.1%, *Gemmiger* 2.9%, *Alistipes* 2.4%, and *Lachnospira* 2.0% were dominant in healthy controls (Figure 1). In the T2D group compared to healthy adults, *Ruminococcus* (*p* < 0.0001), *Blautia* (*p* < 0.001), *Bacteroides* (*p* < 0.001), and *Bifidobacterium* (*p* < 0.05) were predominant at the genus level, and at the strain level, *Ruminococcus bromii* (*p* < 0.0001), *Prevotella copri* (*p* < 0.0001), *Collinsella aerofaciens* (*p* < 0.0001), *Veillonella dispar* (*p* < 0.0001), *Eubacterium bioforme* (*p* < 0.0001), *Dorea formigenerans* (*p* < 0.001), *Lactobacillus ruminis* (*p* < 0.001), *Rothia mucilaginosa* (*p* < 0.0001), and *Gemmiger focilis* (*p* < 0.0001) were higher in relative abundance. In the healthy control group, *Faecalibacterium* at the genus level (*p* < 0.05) and *Ruminococcus callidus*, *Bacteroides uniformis*, and *Faecalibacterium prausnitzii* at the strain level (*p* < 0.05) were statistically significantly higher in relative abundance than they were in the T2D group (Figure 1). The LEfSe analysis showed that, in the T2D group, at the genus level, *Lactobacillus*, *Rothia*, *Collinsella*, and *Eubacterium* increased in relative abundance, and at the species level, the *Rothia mucilaginosa*, *Collinsella aerofaciens*, and *Eubacterium bioforme* strains were found to be dominant.

### 3.2. Comparison of Microbial Compositions in Patients with T2D at Admission and 12 Weeks Later

**Figure 2:**
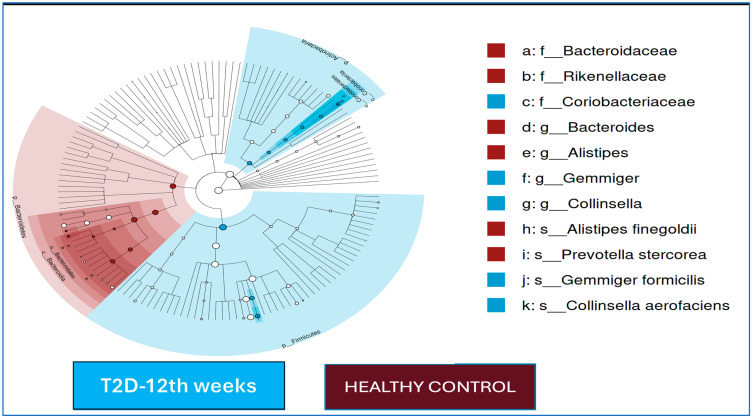
LEfSe analysis results: A cladogram showing that each link of the radial phylogenetic tree is a different taxonomic level in stool samples for the T2D group and healthy controls at 12 weeks of intervention. Horizontal bars represent log_10_-transformed LDA scores indicated by vertical dotted lines. Maroon dots indicate T2D cases, and no imprinting microorganisms were detected in healthy adults in the LEfSe analysis. Maroon: healthy controls. Blue: T2D after 12 weeks of treatment.

In the T2D group, no statistically significant differences were found for the alpha and beta diversity indices at baseline and at the end of the 12th week (*p* > 0.05 for all). While *Faecalibacterium* at the genus level and *Faecalibacterium prausnitzii* at the strain level increased in relative abundance in the T2D group after 12 weeks of follow-up, the *Succinovibrio* genus was more dominant at the beginning of this study compared to the end of the 12th week. The LEfSe analysis results at baseline and at the end of the 12th week in T2D patients are shown in Figure 2.

### 3.3. Comparison of Microbial Compositions of T2D Patients and Healthy Controls at 12-Week Follow-Up

The median values of the Chao1 index, Shannon index, and observed number of OTUs were significantly lower in the T2D group compared to the control group at the beginning of this study and at the end of the 12th week (*p* < 0.01 for all three indices). There were no significant differences in the beta diversity index results (*p* > 0.05).

The LEfSe analysis results for the T2D cases and healthy controls after 12 weeks of follow-up are shown in Figure 2. At the end of the 12th week, in the T2D group, the *Gemmiger* and *Collinsella* genera were dominant, and *Gemmiger formicilis* and *Collinsella aerofaciens* were dominant at the species level; in the control group, *Bacteroides* and *Alistipes* were dominant at the genus level, and *Prevotella stercorea* and *Alistipes finegoldii* were the dominant species.

### 3.4. Metformin and Linagliptin Groups

At the beginning of treatment, there was no difference between the linagliptin group and the metformin group in terms of their alpha and beta diversity parameters (*p* > 0.05). In the linagliptin group, there was a decrease in the median values of the Shannon and Chao-1 indices between the baseline and the 12th week (*p* < 0.05 and *p* < 0.01). In the metformin group, there was no difference in the median values of the Shannon and Chao-1 indices between the baseline and the 12th week (*p* > 0.05).

**Figure 3:**
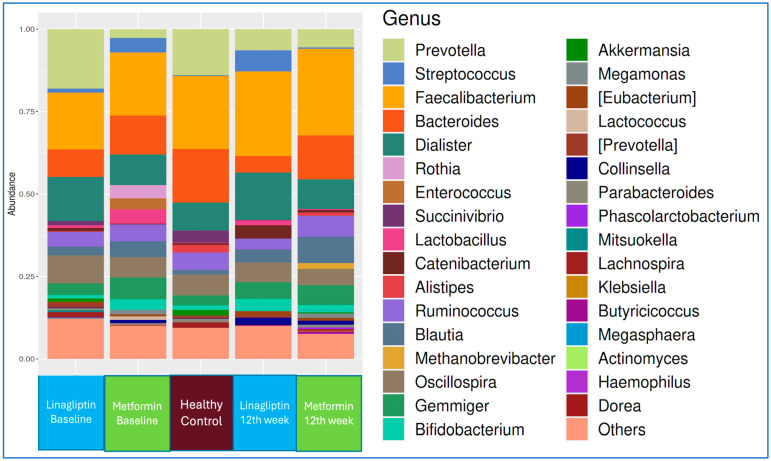
Comparison of the genus-level microbiota compositions of the stool of T2D patients receiving linagliptin (blue) or metformin (green), at the beginning of treatment and after 12 weeks of treatment, with that of healthy controls (maroon).

The genus-level microbiota compositions of the linagliptin and metformin subgroups of T2D patients at the beginning of treatment and at the end of 12 weeks, in comparison with that of healthy controls, are shown in Figure 3.

**Figure 4:**
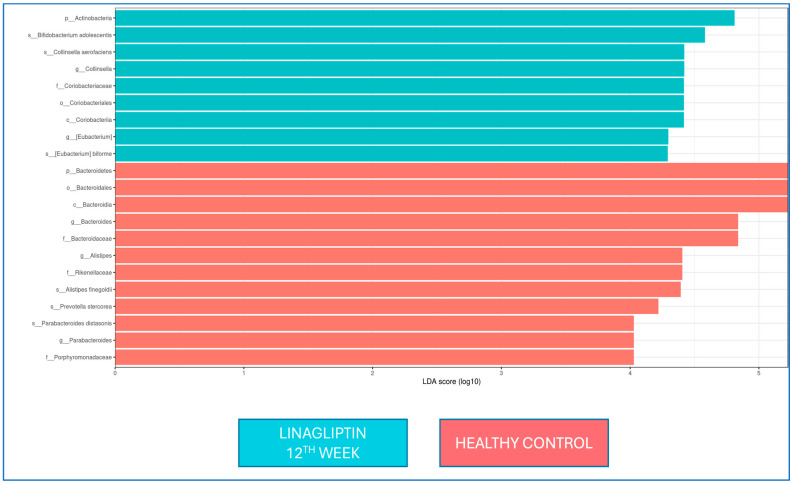
LEfSe analysis of fecal samples from the linagliptin group at Week 12 (green) and healthy controls (red). Horizontal bars represent log_10_-transformed LDA scores indicated by vertical dotted lines. p, family; c, class; o, team; f, family; g, genus; and s, species. (LDA threshold value > 2, *p* < 0.05.).

There was no difference in the LEfSe analysis between baseline and 12 weeks of linagliptin treatment. When the microbiota composition was compared with that of healthy controls at the 12th week of linagliptin treatment, *Bacteroides*, *Alistipes*, and *Parabacteroides* were dominant at the genus level in the linagliptin group, while *Alistipes finegoldii*, *Prevotella stercorea*, and *Parabacteroides distasonis* were dominant at the strain level; in healthy adults, Collinsella and Eubacterium were dominant at the genus level, while *Bifidobacterium adolescentis*, *Collinsella aerofaciens*, and *Eubacterium bioforme* were the dominant strains (Figure 4).

**Figure 5:**
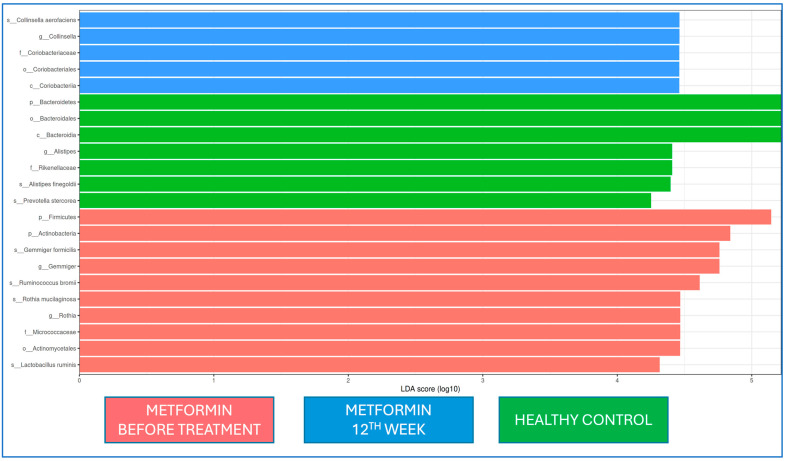
LEfSe analysis of fecal samples from the metformin group at baseline (red) and Week 12 (blue) and from healthy controls (Group 3, green). Horizontal bars represent log_10_-transformed LDA scores indicated by vertical dotted lines. p, family; c, class; o, team; f, family; g, genus; and s, species. (LDA threshold value > 2, *p* < 0.05).

When the microbiota at baseline and 12 weeks of metformin treatment and from healthy controls were evaluated by LEfSe analysis, Firmicutes and Actinobacteria at the phylum level and *Gemmiger* and *Rothia* at the genus level were dominant at the beginning of metformin treatment, while *Coriobactericeae* at the phylum level and *Collinsella* at the genus level were dominant at the end of the 12th week; *Bacteroides* at the phylum level and *Alistipes* at the genus level were dominant in the healthy control group. At the strain level, *Gemmiger formicilis*, *Ruminococcus bromii*, *Rothia mucilaginosa*, and *Lactobacillus ruminis* were dominant at the beginning of metformin treatment, while *Collinsella aerofaciens* was dominant after the 12th week of metformin treatment (Figure 5).

## 4. Discussion

In our study, we found that adult patients with T2D had a different intestinal microbiota composition at the time of diagnosis compared to healthy adults: their bacterial diversity and richness were decreased. After 12 weeks of diet and oral antidiabetic monotherapy (metformin or linagliptin), the patients’ HbA1c levels decreased, and there were some alterations or improvements in the intestinal microbiota composition. However, the microbiota composition remained different in T2D cases compared to healthy individuals. Many studies have shown the role of the microbiota in chronic metabolic diseases such as diabetes and obesity. Although data on whether the microbiota is the cause or effect of these diseases are still being evaluated, microbiota composition has been shown to play an important role in the development of T2D and its prognosis [25].

Research on T2D has demonstrated a disturbance in the composition of the intestinal microbiota. Our study revealed that Firmicutes was the most prevalent phylum in both the patient and control groups. However, Firmicutes was more common in healthy adults, while *Bacteroidetes* was more common in people with T2D than in healthy adults. In our study, at the genus and species levels, there were significant differences between patients with T2D and healthy controls. *Ruminococcus*, *Blautia*, *Bacteroides*, and *Bifidobacterium* at the genus level and *Ruminococcus bromii*, *Prevotella copri*, *Collinsella aerofaciens*, *Veillonella dispar*, *Eubacterium bioforme*, *Dorea formigenerans*, *Lactobacillus ruminis*, *Rothia mucilaginosa*, and *Gemmiger focilis* at the species level were common in patients with T2D, while at the strain level, *Faecalibacterium prausnitzii*, *Ruminococcus callidus*, and *Bacteroides uniformis* were common in healthy adults. Gurung et al. [26] conducted a systematic review and found that the gut microbiome of T2D subjects consistently showed a decrease in relative abundance of the genera *Bifidobacterium*, *Bacteroides*, and *Akkermansia*, along with a consistent increase in relative abundance of the genera *Ruminococcus*, *Fusobacterium*, and *Blautia*. Larssen et al. [27] found a connection between serum glucose levels and the Bacteroidetes-to-Firmicutes ratio, as well as the *Bacteroides*–*Prevotella* to *Clostridium coccoides*–*Eubacterium rectale* ratio, in patients with T2D. Previous studies have shown that butyrate-producing microorganisms, like *Eubacterium rectale*, *Faecalibacterium prausnitzii*, *Roseburia intestinalis*, and *R. inulinivorans*, are less common in people with T2D [28]. In Korea, a study on identical twins reported the potential use of *Akkermansia muciniphila* as a parameter for early diagnosis of T2D [29]. Reports also suggest that diabetes may be associated with the *Acidaminococcus*, *Aggregatibacter*, *Anaerostipes*, *Blautia*, *Desulfovibrio*, *Dorea*, and *Faecalibacterium* genera [30]. The mild decrease in alpha diversity observed in our study, both at the time of diagnosis and 12 weeks later, suggests that diabetes is associated with a moderate level of inflammation. In our study, after 12 weeks of medical treatment and diet, we saw a positive change in the microbiota composition. There was an increase in *Faecalibacterium* at the genus level and *Faecalibacterium prausnitzii* at the strain level. The blood sugar levels also stayed the same. The fact that alpha diversity indicators remained low at the end of the 12th week in T2D cases, the prevalence of the *Gemmiger* and *Collinsella* genera in the T2D group, and the dominance of *Gemmiger formicilis* and *Collinsella aerofaciens* at the species level demonstrated that the microbiota composition was still different to that of healthy controls. Previous studies indicated *C. aerofaciens* as a pathobiont that is also overabundant in metabolic syndrome, T2D, autoimmune polyendocrine syndrome type 1, and psoriatic arthritis. Our results highlighted that some predominant bacteria remained predominant at the 12th week of intervention, despite some improvement in HbA1c levels [31]. Hur and colleagues [32] showed that *Gemmiger formicilis* and *Collinsella aerofaciens* were higher in relative abundance in adults receiving a Westernized diet. Collinsella genus, including *C. aerofaciens*, a trimethylamine (TMA)-producing bacteria with atherogenic effects, was associated with cardiovascular risk in T2D patients [33]. In an experimental model, *C. aerofaciens* is also linked with increased ethanol production and liver inflammation contributes to the pathophysiology of non-alcoholic fatty liver disease [34]. Considering the effects of many factors on diabetes and its course, it seems unlikely that 3 months of diabetes and drug treatment will provide a complete improvement in the microbiota composition, as expected. Improvements in the microbiota composition over time with sustainable lifestyle changes and treatment compliance may be among the long-term treatment goals in these patients.

Metformin is the most common and first recommended oral antidiabetic drug for the treatment of T2D. Experimental and clinical studies have shown that the benefits of metformin are, at least partially, attributable to its impact on gut microbiota [35]. Metformin specifically enhances the prevalence of mucin-degrading *Akkermansia muciniphila* and short-chain fatty acid (SCFA)-producing genera, including *Megasphaera* and *Blautia*, thereby augmenting the advantageous effects of mucin and SCFAs on intestinal barrier integrity, bile acid metabolism, and glucose homeostasis. Metformin further enhances the prevalence of *Bifidobacterium bifidum*. Researchers have proposed that the confounding effects of metformin may account for the heightened prevalence of *Lactobacillus* species. Previous studies suggested that these bacteria could enhance the effects of metformin by producing more butyrate and propionate, and they hypothesized that an excess of *Escherichia coli* species in the gut could contribute to an inability to metabolize metformin [5,10,36]. Studies suggest that metformin benefits microbiota both directly and by regulating blood glucose [10,37,38,39]. Researchers suggest that the positive impact of metformin on the microbiota in T2D patients may stem from its beneficial effects on short-chain fatty acids like butyrate and propionate, which stimulate intestinal gluconeogenesis [10,12]. Researchers also suggest that stimulating gluconeogenesis in the intestines reduces appetite by influencing glucose production in the liver, potentially leading to positive effects on weight loss and metabolic control [40,41]. Studies have also shown that T2D patients who are not taking metformin have fewer microbial genes associated with glycine and tryptophan breakdown than patients who are taking metformin [42]. In our study, 12 weeks of metformin treatment had some beneficial effects on microbiota composition.

DPP-4 inhibitors are another group of oral antidiabetic drugs used in the treatment of T2D. Studies have shown changes in the gut microbiota with DPP-4 inhibitors. Unlike other DPP-4 inhibitors, linagliptin undergoes 90% elimination in feces following oral administration, rather than in the kidneys. The enterohepatic system converts a small portion of absorbed linagliptin to its pharmacologically inactive metabolite. Linagliptin binds 75–99% to plasma proteins and has a half-life of 12 h [43,44]. Researchers found that the DPP-4 inhibitors sitagliptin and vildagliptin alter the gut microbial composition in diabetic rats [45,46,47]. A study using vildagliptin, a DPP-4 inhibitor, revealed a decrease in *Oscillibacter* and an increase in *Lactobacilli*. Olivares et al. [46] suggested that inhibition of DPP-4 may have a direct effect on the gastrointestinal tract. Smith et al. [16] evaluated the effect of sitagliptin on intestinal microbiota composition. They added sitagliptin (100 mg) to metformin and/or sulfonylureas treatment in 19 adult patients with T2D and showed that sitagliptin had no effect on the alpha or beta diversity of the intestinal microbiota, nor were there any changes in microbial composition related to clinical parameters. Our study is a pivotal study showing the effect of linagliptin on microbiota composition. We found no changes in microbiota composition (with LEfSe analysis) between the baseline and 12th week of intervention in the linagliptin group. Regarding the abundance in *F. prausnitzii*, there is an increase (from 32% to 48%) at 12 weeks of linagliptin use, there are no statistical differences, and the low sample size might affect these results. When the microbiota composition was compared with that of healthy controls at the 12th week of linagliptin treatment, *Bacteroides*, *Alistipes*, and *Parabacteroides* were dominant at the genus level, while *Alistipes finegoldii*, *Prevotella stercorea*, and *Parabacteroides distasonis* were dominant at the strain level in the linagliptin group.

This study had some limitations. It is difficult to control each parameter related to microbiota composition in patients with T2D. Our enrollment number was considerably low; however, we enrolled patients receiving only monotherapy and excluded those with different clinical conditions related to T2D. Due to the study design and the nature of the disease, this study was not a placebo-controlled trial. While the patients received dietary and exercise advice, this study lacked any control or documentation of dietary intake, which can strongly influence gut microbiota composition. We did not show any correlation between HbA1c levels and alpha diversity indices; this might be related to the short course of therapy and needs to be evaluated in further studies.

## 5. Conclusions

In conclusion, we observed a substantial change in the composition of the gut microbiota in patients with type 2 diabetes. Twelve weeks of an oral antidiabetic treatment, especially metformin, had some beneficial effects on microbiota composition. Limited research has investigated the relationship between antidiabetic medications and the gut microbiota. More research needs to be conducted to fully understand how these drugs change the microbiota in the gut and how such changes help to improve glucose homeostasis.

## Data Availability

Data available on request from the authors.
